# Investigating the antioxidant activity enhancer effect of *Cyamopsis tetragonoloba* seed extract on phenolic phytochemicals

**DOI:** 10.3389/fpls.2023.1131173

**Published:** 2023-03-08

**Authors:** Tripti Joshi, Sumit Kumar Mandal, Sonakshi Puri, Vidushi Asati, P. R. Deepa, Pankaj Kumar Sharma

**Affiliations:** Department of Biological Sciences, Birla Institute of Technology and Science (BITS), Rajasthan, India

**Keywords:** *Cyamopsis tetragonoloba*, phenolics, antioxidant activity enhancer, cytoprotective effect, lipid peroxidation, nutraceutical

## Abstract

**Introduction:**

Phenolic phytochemicals are known for antioxidant-mediated pharmacological effects in various diseases (diabetes, cancer, CVDs, obesity, inflammatory and neurodegenerative disorders). However, individual compounds may not exert the same biological potency as in combination with other phytochemicals. *Cyamopsis tetragonoloba* (Guar), an underutilized semi-arid legume which has been used as a traditional food in Rajasthan (India), is also a source of the important industrial product guar gum. However, studies on its biological activity, like antioxidant, are limited.

**Methods:**

We tested the effect of *C. tetragonoloba* seed extract to enhance the antioxidant activity of well-known dietary flavonoids (quercetin, kaempferol, luteolin, myricetin, and catechin) and non-flavonoid phenolics (caffeic acid, ellagic acid, taxifolin, epigallocatechin gallate (EGCG), and chlorogenic acid) using DPPH radical scavenging assay. The most synergistic combination was further validated for its cytoprotective and anti-lipid peroxidative effects in *in vitro* cell culture system, at different concentrations of the extract. LC-MS analysis of purified guar extract was also performed.

**Results and discussion:**

In most cases, we observed synergy at lower concentrations of the seed extract (0.5-1 mg/ml). The extract concentration of 0.5 mg/ml enhanced the antioxidant activity of Epigallocatechin gallate (20 µg/ml) by 2.07-folds, implicating its potential to act as an antioxidant activity enhancer. This synergistic seed extract-EGCG combination diminished the oxidative stress nearly by double-fold when compared with individual phytochemical treatments in *in vitro* cell culture. LC-MS analysis of the purified guar extract revealed some previously unreported metabolites, including catechin hydrate, myricetin-3-galactoside, gossypetin-8-glucoside, and puerarin (daidzein-8-C-glucoside) which possibly explains its antioxidant enhancer effect. The outcomes of this study could be used for development of effective nutraceutical/dietary supplements.

## Introduction

Legumes, or dry beans and pulses, are members of the Fabaceae family that grow in pods of annual, biennial, and perennial plants. They are not only one of the largest but also among the economically most significant families of flowering plants due to their nitrogen-fixing capacity and restoration of nitrogen-depleted soil by crop rotation (Mbagwu et al., 2011). Legumes are generally recognized for their high concentrations of bioactive components, including phenolics, phytosterols, carbohydrates, and saponins, which help lower the risk of oxidizing substances, bacteria, diabetes, inflammatory disease, and cancer (Ayilara et al., 2022; Jat et al., 2023). Numerous research endeavours have discovered the antioxidant properties of different species of legumes, and they have found a strong correlation between antioxidant potential and total phenolic content (Asati et al., 2022; Timoracká et al., 2022). At a time when one out of every five children under the age of five is chronically malnourished, legumes are now considered a future superfood capable of eradicating hunger and contributing to health (Martín-Cabrejas, 2018; Chaudhary et al., 2022). Due to their low cost and positive environmental impact, their natural bioactive compounds are currently a trend in the food processing industry (Riaz et al., 2022). As consumers become more aware of the nutritional and nutraceutical composition of legumes, their global demand continues to rise (Pham and Luan, 2021).

**Figure 1:**
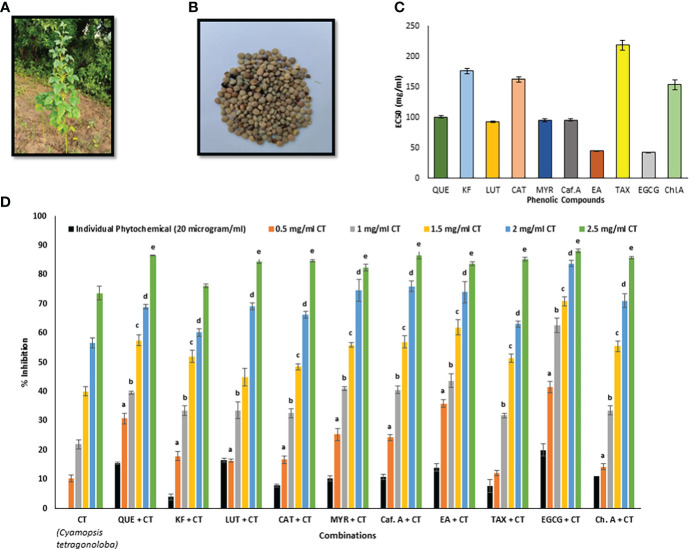
**(A)** *C. tetragonoloba* plant, **(B)** *C. tetragonoloba* seeds, **(C)** EC_50_ values of individual phenolic compounds, **(D)** DPPH inhibition% of phenolic compounds with and without *C. tetragonoloba* seed extract. CT, *C. tetragonoloba*; QUE, quercetin; KF, kaempferol; LUT, luteolin; CAT, catechin; MYR, myricetin; Caf. A, caffeic acid; EA, ellagic acid; TAX, taxifolin; EGCG, epigallocatechin gallate; Ch. A, chlorogenic acid. Values denote mean ± SD (n=3). a, b, c, d, and e represent statistically significant different values (P ≤ 0.05) with respect to 0.5, 1, 1.5, 2, & 2.5 mg/ml concentration of CT, respectively.

*Cyamopsis tetragonoloba* (Guar), has been used traditionally for food and fodder purposes (Pankaj and Dhankar, 2023) (**Figures 1A, B**). It is economically important and also known as the heart of the farmer fields, as India contributes 80% of the global guar gum production. Guar can be used as a laxative, digestive aid, appetizer, or cooling agent (Mukhtar et al., 2004). Potentially, guar gum can help hypercholesterolemic insulin-dependent diabetic patients with improved glycemic control and lower serum LDL-cholesterol concentrations (Vuorinen-Markkola et al., 1992). Potent phytochemicals including phenolics and flavonoids are found in the seeds (Kuravadi et al., 2012). Owing to the presence of multiple therapeutically active molecules, like quercetin, daidzein, and kaempferol, it is used as a complementary medicinal plant (Jain and Rijhwani, 2018). For example, Kaushik et al. (2020) reported that *C. tetragonoloba* could play an important role in developing inexpensive and effective anti-dengue medicine.

Plant secondary metabolites are multifunctional metabolites produced from various biochemical pathways. The biosynthesis of aromatic amino acids, tryptophan, tyrosine, and phenylalanine, which are common precursors for phenolics and nitrogen-containing compounds is initiated by the shikimate pathway (Jan et al., 2021; Elhamouly et al., 2022). As per García-Calderón et al. (2020), the secondary metabolites originating from the phenylpropanoid metabolism include monolignols, flavonoids and isoflavonoids, various phenolic acids, and stilbenes, which function to: protect the plants against oxidative stress and pathogens; also as chemical signals in symbiotic nitrogen fixation with rhizobia. Further, despite the important role played by the *Lotus japonicus* (a model legume) in elucidating the molecular genetics of legume–rhizobia symbiosis, the class of phenolic compounds used by this species in order to attract its chosen symbiont is still unknown. Species-specific differences in flavonoid accumulation have also been observed. For example, in *L. japonicas*, different types of abiotic stress situations (such as UV-B irradiation) resulted in an accumulation of isoflavonoids as a possible alternative to accumulation of flavonols.

Phenolics are a broad class of bioactive compounds that contain at least one benzene ring and one or more hydroxyl groups. The complexity of phenolic compounds ranges from simple phenols to highly polymerized compounds (Lin et al., 2016). These compounds are differentially distributed in the cotyledon (mainly non-flavonoid phenolics) and the seed coat (flavonoids) of legumes (Amarowicz, 2020). The distinctive bioactive potential, color and flavor of legumes are due to the most abundant phenolic compound, flavonoids, which are composed of two aromatic rings linked by a 3-C bridge, in the form of heterocyclic C ring (Pham and Luan, 2021).

In humans, oxidative stress is caused by an imbalance between the formation of reactive oxygen species (ROS) and the endogenous antioxidants, leading to a reaction cascade that can damage lipids, proteins, and DNA (Rudrapal et al., 2022). Antioxidants operate as scavengers of reactive free radicals, inhibiting lipid peroxidation and other related processes, and thereby protect the body from resulting diseases (Pande and Srinivasan, 2013b). The antioxidant properties of phenolic compounds are influenced by their chemical makeup. The most important aspects of flavonoids’ activity as main antioxidants are the position and degree of hydroxylation on the B ring (Kerwin, 2004).

According to the reports by World Health Organisation (WHO), nearly 80% of the global population depends on plant based medicines owing to their positive impact on health and lower side-effects (Adki et al., 2020; Singh and Gaikwad, 2020). There is a plethora of research stating the antioxidant potential of individual pure phytochemicals or plant extracts. However, despite knowing their excellent antioxidant activity and natural origin, studies on the biological activities of their combinations are surprisingly limited. It is reasonable to hypothesize that edible plant extracts can be used to enhance the antioxidant activity of known phytochemicals, thereby increasing their bioactive potential. The current research focuses on investigating the antioxidant activity enhancer (AAE) effect of *Cyamopsis tetragonoloba* seed extract on dietary flavonoids (quercetin, kaempferol, luteolin, myricetin, and catechin) and non-flavonoid phenolics (caffeic acid, ellagic acid, taxifolin, epigallocatechin gallate (EGCG), and chlorogenic acid) using DPPH radical scavenging assay and lipid peroxidation assessment in *in vitro* cultured cells. The total phenolic content and total flavonoid content were calculated. LC-MS analysis was performed to identify possibly novel and unreported compounds (also as potential contributors to antioxidant activity enhancement of standard phenolic phytochemicals) from the seed extract of *C. tetragonoloba*.

## Materials and methods

### Chemicals and reagents

Quercetin, kaempferol methanol, dimethyl sulfoxide (DMSO), DPPH, Folin Ciocalteu reagent, sodium carbonate, aluminum chloride, sodium nitrite, sodium hydroxide, Amberlite XAD7HP, Sephadex LH-20 were procured from Sigma-Aldrich Chemicals Company (United States). Luteolin, catechin, myricetin, caffeic acid, ellagic acid, taxifolin, epigallocatechin gallate (EGCG), chlorogenic acid, gallic acid were obtained from Yucca Enterprises (Mumbai, India), Mouse embryonic fibroblast cells (3T3-L1) were obtained from National Centre for Cell Science (NCCS, Pune, India), Dulbecco’s Modified Eagle’s Medium (DMEM) and fetal bovine serum (FBS) were purchased from Gibco Life Technologies (Carlsbad, CA, USA), 2-Thiobarbituric acid (TBA), Trichloroacetic acid (TCA) were purchased from Sigma Aldrich.

### Plant collection and extraction

Commercially available seeds of *Cyamopsis tetragonoloba* (guar) were purchased from a local grocery store in Pilani market (Jhunjhunu district, Rajasthan, India).

### Preparation of seed extract

The seeds of *Cyamopsis tetragonoloba* were ground to a fine powder using a Waring blender. The fine powder was defatted with hexane in a 1:5 (w/v) ratio at room temperature for 1 hr in an orbital shaker incubator. This was followed by centrifugation at 3000xg for 10 min. The supernatant was decanted and the pellet was extracted two more times. The hydrophobic compounds were separated in the hexane extract and the pellet was dried at room temperature. This dried pellet was extracted thrice with 5 volumes of 80% methanol by the same process mentioned above. The supernatant was filtered using a Whatman filter paper (No. 1). This step was repeated twice, and all the supernatants were pooled and concentrated to dryness using a rotary evaporator (Aditya Scientific, Hyderabad, India). The concentrated extracts were stored at 4°C for further analysis.

### Total phenolic content

The TPC was determined by the Folin-Ciocalteu method described by (Slinkard and Singleton, 1977; Tsao et al., 2003; Wang et al., 2011) with slight modifications. Briefly, 200 µl of the seed extract was mixed with 800 µl of 7.5% sodium carbonate and 1ml of the FCR (Folin-Ciocalteu Reagent). The mixture was shaken gently and incubated at room temperature for 30 min, and the absorbance was read at 765 nm. A gallic acid standard curve was prepared with different concentrations (50-250 μg/ml), and the TPC values were expressed as micrograms of gallic acid equivalents (GAE) per gram of sample. All tests were performed in triplicates.

### Total flavonoid content

The TFC was calculated by the Aluminum chloride colorimetric assay described by John et al. (2014). In brief, an aliquot (1ml) of extracts or standard solutions of quercetin (200-1000 μg/ml) was added in a flask containing 4 ml distilled water. To the flask was added 300 µl 5% NaNO_2_, followed by 300 µl 10% AlCl_3_ after five minutes. This was followed by addition of 2ml 1M NaOH, and the volume was made up to 10 ml with distilled water. The solution was shaken, and absorbance was read at 510 nm. The TFC values were expressed as mg of quercetin equivalents per g of sample. All tests were performed in triplicates.

### Estimation of antioxidant potential

The antioxidant activity of plant seed extract and pure phytochemicals was tested by DPPH (2,2-diphenylpicrylhydrazyl) assay (after 2-fold dilution). For binary combinations (to test the potential enhancement of the antioxidant activity of pure phytochemicals by *C. tetragonoloba* seed extract), the seed extract was used in varying concentrations (0.5-2.5 mg/ml) whereas the concentration of pure compounds was kept constant (20 µg/ml). These were mixed in a 1:1 (v/v) ratio.

### DPPH free radical scavenging assay

The DPPH assay was performed as described by Hidalgo et al. (2010). The methanolic solution of DPPH is purple/violet colored, which fades to pale yellow in the presence of antioxidants, and the loss in absorbance is measured at 517 nm. A 100 μM DPPH solution was prepared in methanol, and 290 μl of this solution was mixed with 10 μl of individual compound/seed extract or their combinations. The reaction was carried out in a 96-well microplate, incubated in the dark at room temperature for 1hr, and absorbance was measured at 517 nm using a microplate reader (ThermoScientific Multiskan G0). The percentage DPPH radical scavenging activity was calculated by the following equation:


$$Inhibition\;\%=\frac{A_{c}-\;A_{s}}{A_{c}}$$

Where A_c_ is the absorbance of the control and A_s_ is the absorbance of the sample. Solution without the sample (seed extract or phytochemical) was taken as control. The results were expressed as EC_50_ (μM) obtained by plotting a curve between concentration and inhibition percentage. EC_50_ is the effective concentration necessary to get 50% inhibition. The lower the EC_50_ value, higher will be the antioxidant activity.

### Cell viability assay

The non-toxic dosage of test phytochemicals - CT and EGCG, was determined by MTT (3-(4, 5-dimethylthiazolyl-2)-2, 5-diphenyltetrazolium bromide) based cell viability assay, which shows the metabolic activity of cells. Murine fibroblast NIH-3T3 cells (NCCS, Pune, India) were cultured at 37°C in humidified 5% CO_2_ in Dulbecco’s modified eagle medium (DMEM) supplemented with 10% (vol/vol) fetal bovine serum (FBS), penicillin G and streptomycin (100 mg/l). After the cells were confluent, cells were trypsinized from the surface of the culture flask by using a 0.25% trypsin solution. The cells were plated on the cultivation flask (surface 25 cm^2^) at a density of 6 X 10^4^ NIH-3T3 cells per ml medium and incubated for 24 h prior to the experiments. The NIH/3T3 cells were seeded in a 96-well (8 × 10^3^ cells per well) and were incubated for 24 h. The test phytochemicals, CT and EGCG, were dissolved in DMSO (stock solution 10 mM), and diluted in media to a final concentration of 5 µM to 50 µM (concentration of DMSO 0.5%). After 24 h of incubation, 90 µl of growth medium and 10 µl MTT dye (5 mg/ml) were added to each well and incubated for an additional three hours. The MTT solution-containing media was then removed. After adding DMSO the plate was shaken gently to dissolve the formazan crystals. The absorbance was measured at 570 nm (Multiskan FC, Thermo Scientific, DE) (Ahmad et al., 2019; Mandal et al., 2022). The percentage of cytotoxicity was determined as follows:


% cell viability=(Mean absorbance of treated group)/(Mean absorbance of control group)×100

### Lipid peroxidation assay

To evaluate the effect of phytochemicals (individual and in combination) on oxidative stress, levels of malondialdehyde (MDA), a stable end product of lipid peroxidation was estimated by TBARS (thiobarbituric acid reactive substances) assay. NIH/3T3 cells without any treatment with H_2_O_2_ or phytochemicals served as the control, while the fibroblast cells exposed to 100 µM H_2_O_2_ for 6 hrs served as the oxidative stress induced model. NIH/3T3 cells were initially treated with the phytochemicals for 3h, followed by exposure to 100 µM H_2_O_2_. After 3h, the cells were treated with lysis buffer, homogenized, centrifuged at 13000 x g at 4°C for 15 minutes and the supernatant was collected. Cell lysates of each experimental group were normalized on the basis of equal amount of protein (100 µg), and incubated with 500 µl of 10% trichloroacetic acid (TCA). This was followed by reacting with 750 µl of thiobarbituric acid (TBA, 1% w/v) in an acidic condition, and the solution was heated in a boiling water bath for 15 minutes to generate a pink colour adduct which was measured spectrophotometrically (Multiskan FC, Thermo Scientific, DE) at 530 nm (Wenz et al., 2019). Values were expressed as µM of malondialdehyde/mg protein.

### Purification of phenolics and flavonoids from *C. tetragonoloba* seed extract

In order to identify potential phytocompounds in the *C. tetragonoloba* seed extract leading to antioxidant activity enhancement of the pure phytochemical, the seed extract was subjected to column chromatography. Purification was done by previously reported protocol with slight modifications (Asati et al., 2022). In brief, Amberlite XAD7HP chromatography followed by Sephadex LH-20 were used to purify the defatted methanol extract. 5 gm of the extract was loaded on the matrix packed in a glass column (50 cm x 1.8 cm) and equilibrated using 100% methanol. Initially, 50% methanol was used to elute the column, and 20 fractions of 5 mL each were collected. This was followed by elution using 100% methanol. All fractions were analyzed by TLC (Thin layer chromatography) on silica gel F254 plates (Merck, USA) using Toluene: Acetic Acid: Acetone: Formic Acid::20:4:2:1 as solvent system and plates were visualized at 254 and 366 nm. The fraction exhibiting the maximum bands (C5) was further characterised using HPLC and LC-MS.

### HPLC and LC-MS analysis of the purified fraction

The purified fractions were filtered by 0.45 µm Puradisc filters and HPLC (Shimadzu Corporation, Tokyo, Japan) analysis was performed for the purpose of optimization. Photo diode array detector was used; absorbance was monitored from 200 to 365 nm. The protocol reported by Song et al. (1998) for chromatographic separation was standardized by slight modifications. In brief, separation was performed on a C18 column (SpherisorbR, 250 mm x 4.6 mm, particle size 5 µm, Waters) with optimized mobile phase A (pH 3.0 Milli Q water) and B (Acetonitrile). Glacial acetic acid was used as pH modifier. The system was equilibrated for an hour at 1 ml/min flow rate. Initially, phase A and acetonitrile concentrations were 10% and 90%, respectively, for the purpose of washing. After sample injection, 0.1% glacial acetic acid in acetonitrile was maintained at 15% for 5 minutes. Solvent B reached 35% in 33 minutes and 10% in 40 minutes. The solvent flow rate was l mL/min for the first 5 min, increased to 1.5 mL/min over 0.5 min and maintained for 39 min, and then returned to 1 mL/min.

The C5 fraction obtained through Sephadex LH-20 chromatography (obtained as mentioned in the previous section on purification) was further analysed by LC-MS. This was performed as per Chaudhary et al. (2020) and Venuprasad et al. (2014). Q-TOF Micromass spectrometer (Waters Corporation, Milford, MA, USA) was used. Chromatographic separation was done using Spherisorb 5 µm ODS2 column with the help of auto sampler (flow rate of 0.2 mL/min, 280 nm wavelength and 20 μL injection volume). Solvents were: (A) Formic acid (0.1% v/v) and 10 mM ammonium fluoride and (B) acetonitrile + 0.1% Formic acid. Gradient (in solvent B) was: (i) 30%, from 0 to 15 min, (ii) 55%, from 15 min, (iii) 95%, from 25 to 45 min, and (iv) 35%, at 45–48 min; spray voltage 4 KV; gas temperature 325°C; gas flow 10 L/min; and nebulizer 40 psi. Electrospray mass spectra data were recorded on positive and negative ionization mode for a mass range *m/z* 50–*m/z* 1000. The instrument’s MassLynx database was used to examine the products. RIKEN-RESPECT was used to evaluate mass spectrum fragments (Sawada et al., 2012).

### Statistical analysis

Experiments were done in triplicate, and the values were calculated as mean ± standard deviation. One-way analysis of variance (ANOVA) was performed to assess the statistically significant difference between the mean values. P-value ≤ 0.05 was considered statistically significant.

## Results and discussion

### Total phenolic content and total flavonoid content

The antioxidant activity of plants is directly proportional to theirphenolic/flavonoid content (Joshi et al., 2022). It has been reported that phenolic compounds are best extracted with methanol (80% concentration) as compared to other solvents due to its polarity and solubility of phenolics (Moteriya, 2015). The TPC and TFC of the defatted methanolic extract of *C. tetragonoloba* seeds were 280 ± 9.5 mg GAE/g and 496 ± 15.2 mg QE/g of the extract, respectively. Various factors, including growth and storage conditions (climate, soil, water), and time of harvest are responsible for different phytochemical composition (Wright et al., 2001). Our research group has earlier reported the variation in total flavonoid content of *P. cineraria* pod extracts obtained from trees in different geographical regions (Asati et al., 2022). Sharma et al. (2017) reported that the TPC and TFC of different guar cultivars collected from various states of India ranges between 60.03 to 204.67 mg GAE/g and 4.26 to 12.43 mg QE/g, respectively. Thus, cultivar selection is important for functional food development from traditional plants.

### Antioxidant activity of standard phenolic compounds

The antioxidant activity of standard phenolics were tested by DPPH free radical scavenging assay. EGCG (EC_50_- 42.69 ± 0.16 µg/ml) and ellagic acid (EC_50_- 45.08 ± 0.40 µg/ml) showed the maximum antioxidant activity among the 10 tested compounds (**Figure 1C**). The order of antioxidant activity from lowest to highest was as follows- taxifolin< kaempferol< catechin ≤ chlorogenic acid< quercetin< myricetin = caffeic acid< luteolin< ellagic acid ≤ EGCG. This result is in accordance with the trend of antioxidant activity reported by other researchers (Hirano et al., 2001; Hidalgo et al., 2010). It is believed that the gallate group at position 3 plays the most crucial role in their ability to scavenge free radicals, with an extra hydroxyl group inserted at position 5’ in the B ring also contributing to their scavenging capabilities (Heim et al., 2002; Braicu et al., 2011).

Arrangement and number of hydroxyl moieties on the ring, presence of catechol group in the B ring, and 2, 3 double bonds in the C ring, are some characteristics that strongly correlate with antioxidant potential. According to Freeman et al. (2010), these groups can also be used to find the reduction potentials, as a molecule with lower reduction potential has more tendency to donate its electron and act as a strong antioxidant. The results are in accordance with these reports; Quercetin, myricetin, and luteolin showed almost similar antioxidant activities because of almost similar structure (a catechol group in the B ring and a 2, 3 double bond in the C ring (Freeman et al., 2010)) while EGCG has the lowest reduction potential, thereby showing maximum activity.

### Antioxidant activity enhancer effect of *C. tetragonoloba* seed extract on standard phenolic phytochemicals

*C. tetragonoloba* seed extract was used in combination with 10 phenolic compounds to test for possible synergism (antioxidant activity enhancement). The DPPH radical scavenging activity of the pure compounds with and without the seed extract were compared (**Figure 1D**). It was observed that with increase in the concentration of extract from 0.5-2.5 mg/ml, the DPPH % inhibition also increases. **Table 1** shows the percentage inhibition of the combinations at different concentrations and the types of interaction. An interaction can be said to be synergistic when the experimental value is greater than the theoretical value (calculated by summing up the inhibition percentage pertaining to antioxidant activity of individual phytochemicals and seed extracts), additive when the experimental and theoretical values are equal; and when experimental value is less than the theoretical value, it is an antagonistic interaction.

**Table 1:** DPPH inhibition % of all phytochemicals (each at 20 µg/ml) in combination with different concentrations of the *C. tetragonoloba* (CT) seed extract (0.5-2.5 mg/ml).

Combinations		DPPH Inhibition % at different concentrations (mg/ml) ± STDEV									
		0.5		1		1.5		2		2.5	
QUE + CT	E	30.78 ± 1.81^*^	Syn	39.67 ± 0.46^*^	Syn	57.55 ± 1.84	Ad	69.02 ± 0.76^*^	An	86.55 ± 0.15^*^	An
	T	25.94 ± 0.39		37.53 ± 1.33		55.68 ± 2.11		72.19 ± 2.57		89.28 ± 2.40	
KF + CT	E	17.90 ± 1.55^*^	Syn	33.49 ± 1.67^*^	Syn	52.06 ± 2.04^*^	Syn	60.22 ± 1.36	Ad	73.25 ± 0.67^*^	An
	T	14.30 ± 1.19		25.89 ± 1.25		44.04 ± 1.93		60.55 ± 1.32		77.64 ± 2.71	
LUT + CT	E	16.40 ± 0.55^*^	An	33.59 ± 2.91^*^	An	44.94 ± 2.98^*^	An	69.20 ± 1.14^*^	An	84.64 ± 0.77^*^	An
	T	26.99 ± 0.24		38.58 ± 0.99		56.73 ± 2.15		73.24 ± 2.17		90.33 ± 2.61	
CAT + CT	E	16.74 ± 1.31	Ad	32.59 ± 1.67	Ad	48.52 ± 1.10	Ad	66.26 ± 1.12	Ad	84.87 ± 0.38^*^	Syn
	T	18.24 ± 1.43		29.83 ± 2.04		47.98 ± 0.96		64.49 ± 2.25		81.58 ± 1.66	
MYR + CT	E	25.33 ± 2.16^*^	Syn	41.14 ± 0.64^*^	Syn	55.98 ± 0.69^*^	Syn	71.68 ± 3.77	Ad	80.51 ± 1.09^*^	An
	T	20.75 ± 0.94		32.34 ± 1.58		50.49 ± 1.45		67.00 ± 2.12		84.09 ± 2.02	
Caf. A + CT	E	24.31 ± 1.04^*^	Syn	40.61 ± 1.36^*^	Syn	57.01 ± 2.07^*^	Syn	76.04 ± 1.90^*^	Syn	86.72 ± 1.20	Ad
	T	21.18 ± 0.51		32.77 ± 0.69		50.92 ± 2.32		67.43 ± 1.82		84.52 ± 2.88	
EA + CT	E	35.89 ± 1.33^*^	Syn	43.74 ± 2.37^*^	Syn	61.92 ± 2.57^*^	Syn	73.97 ± 3.71	Ad	86.15 ± 0.70^*^	An
	T	24.39 ± 1.94		35.98 ± 2.39		54.13 ± 0.81		70.64 ± 2.14		87.73 ± 1.77	
TAX + CT	E	12.06 ± 0.88^*^	An	31.88 ± 0.80	Ad	51.49 ± 1.42^*^	Syn	63.07 ± 0.97	Ad	85.36 ± 0.62^*^	Syn
	T	18.01 ± 2.05		29.60 ± 2.75		47.75 ± 0.35		64.26 ± 2.88		81.35 ± 0.99	
EGCG + CT	E	41.58 ± 1.89^*^	Syn	62.67 ± 2.50^*^	Syn	70.91 ± 1.60^*^	Syn	83.82 ± 1.03^*^	Syn	88.20 ± 0.69^*^	Syn
	T	22.37 ± 2.74		33.96 ± 3.54		52.11 ± 0.83		68.62 ± 3.74		85.71 ± 0.20	
Ch. A + CT	E	14.28 ± 1.09^*^	An	33.57 ± 1.51	Ad	55.50 ± 1.93^*^	Syn	71.09 ± 2.38	Ad	85.77 ± 0.44	Ad
	T	21.46 ± 1.84		33.05 ± 2.78		51.20 ± 1.83		67.71 ± 3.76		84.80 ± 1.44	

Expanded forms of the abbreviations for the phytochemicals are given in the legend for **Figure 1**.

E, Experimental value; T, Theoretical value; Syn, Synergistic; Ad, Additive; An, Antagonistic interaction. All experiments were done in triplicates. *P-value ≤ 0.05 was considered statistically significant.

As seen from **Figure 1D**, when the seed extract (varying concentrations) was added to the phenolic compounds (20 µg/ml), most of the combinations showed synergistic antioxidant effect at lower concentrations of extract (0.5-1 mg/ml), while on further increasing the extract concentration, additive (at 1.5 mg/ml) and antagonistic (at 2-2.5 mg/ml) interactions were observed. The results agreed with the previous report that phytochemicals need to be combined in specific ratios to show synergistic effect (Joshi et al., 2022). When the extract concentration was 0.5 mg/ml, the DPPH % inhibition of extract with EGCG was 41.58%, which was 4-folds and 2.07-folds higher than that of seed extract (10.37%) and EGCG (20%). All the concentrations of extract showed synergistic interaction with EGCG. Therefore, this synergistic seed extract-EGCG combination was further validated for its cytoprotective and anti-lipid peroxidative effects in *in vitro* cell culture system. Similar results were reported by Zhang et al. (2016), that mulberry leaf polysaccharides (MLPs) can be used as antioxidant activity enhancers of flavonoids. It was reported that despite having low antioxidant activity themselves, MLPs showed synergistic interaction with flavonoids. In another study, combination of *C. tetragonoloba* with garlic and capsaicin (responsible for pungent flavor of red pepper), offer a significant increase in the antioxidant status (Pande and Srinivasan, 2013a; Pande and Srinivasan, 2013b).

Romano et al. (2009) reported the antioxidant activity enhancing effect of rosemary extract on synthetic antioxidants (butylated hydroxyanisole (BHA) and butylated hydroxytoluene (BHT)). This could give the food sector a strong reason to combine natural and synthetic antioxidants in processed food products to increase storage stability and prevent any potential hazardous effects from using excessive levels of antioxidants. Flavonoids (quercetin, kaempferol, and isorhamnetin) present in the almond skin have been shown to act synergistically with vitamin E and C (Chen et al., 2005). In another study, the combination of longan peel extract (LP), vitamin E, and ascorbyl palmitate (derivative of ascorbic acid) lowered the free radicals in tuna oil, contributing to the antioxidant effect; thus, LP could have an application as a food additive against lipid oxidation in oils (Rakariyatham et al., 2021). This study also offered mechanistic insights into antioxidant synergy among phytochemicals.

As *C. tetragonoloba* and the tested phytochemicals (polyphenolic compounds) have been used for their antioxidant property for many years, the current study suggests potential usage of their combination in order to achieve a greater therapeutic effect. These findings may be helpful for people who want to increase their antioxidant intake – without compromising on safety - as well as for the development of novel medications and functional foods with higher antioxidant potential.

There are different hypotheses for possible mechanisms responsible for the above-mentioned interactions. Synergistic interactions could be due to- a) the regeneration of strong antioxidants by the weaker ones (Marinova et al., 2008), b) formation of stable intermolecular adducts with strong antioxidant activity (Olszowy, 2020), c) the type and concentration of antioxidant (Shi et al., 2007). Hypotheses for antagonistic interactions are- a) regeneration of weaker antioxidants by stronger antioxidants, b) polymerization of antioxidants decreases their activity, c) disappearance of free antioxidant radicals due to irreversible reactions. Further studies need to be conducted to validate the specific mechanism for antioxidant synergism observed between the tested phytochemicals and *C. tetragonoloba* seed extract in the current study.

### Validation of antioxidant activity enhancer effect of CT seed extract towards EGCG in cultured fibroblast cells

#### Non-cytotoxic dosage of phytochemicals determined in NIH/3T3 cells

**Figure 2:**
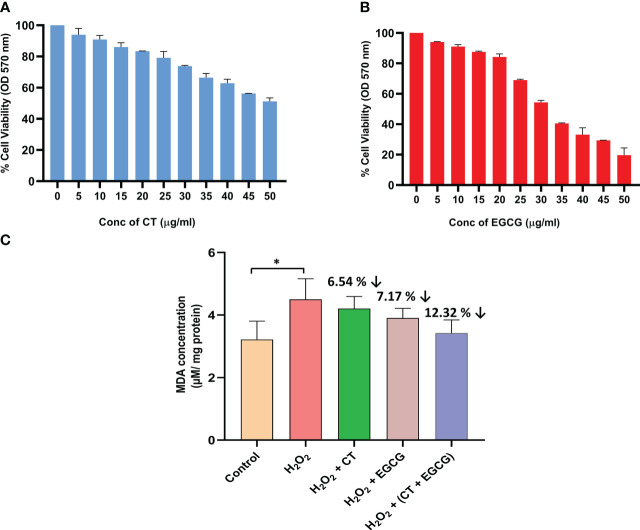
**(A, B)** represent the dose dependent changes in cell viability of normal fibroblast cells exposed to different concentrations of CT and EGCG, respectively. Values denote mean ± SD of three experiments performed in triplicate. **(C)** shows the reduction in oxidative stress (lipid peroxidation) in the H2O2 induced fibroblast cells treated with phytochemicals (20 µg/ml of both CT and EGCG). Values denote mean ± SD of two experiments done in triplicate. The oxidative stress mimic (H2O2 group) and the treated groups were compared with the normal control group where statistically significant difference was expressed at P ≤ 0.05 (denoted by *) (GraphPad Prism v8.0.2).

The viability of the normal fibroblast cells (NIH/3T3) was assessed by MTT assay to evaluate the non-cytotoxic dosage range for CT and EGCG, at various concentrations. The safe dosage of CT, and EGCG was considered to be the concentration at which at least 80% of cells were viable (non-toxic dosage). After 24 hours incubation, the test phytochemicals showed dose-dependent decrease in cell viability, wherein CT and EGCG revealed 80% cell viability upto a dosage of 20 µg/ml (**Figures 2A, B**).

### Lipid peroxidation assay

Following H_2_O_2_ treatment, a significant increment in the concentration of MDA (P ≤ 0.05) was observed in the normal fibroblast cells, indicating that it served as an oxidative stress induced cell culture model. In this induced model, the phytochemicals afforded antioxidant protection to the cells and reduced the MDA levels that were closer to the normal control fibroblast cells. The levels of the biochemical marker of lipid peroxidation, MDA, was decreased by 6.54%, 7.17%, 12.32%, respectively in the H_2_O_2_-induced cells, treated by CT, EGCG, and their combination, respectively. It was interesting to note that the combination of CT and EGCG markedly diminished the oxidative stress nearly by a double-fold when compared with individual phytochemical treatments. This clearly indicated the anti-oxidant enhancer effect of CT on EGCG (**Figure 2C**).

### LC-MS analysis of the enriched and purified fraction C5 obtained from *C. tetragonoloba*

The fractions eluted out of Sephadex LH-20 column were analysed by TLC on Silica gel F254 plates (**Supplementary Material, Figure S2**). One fraction, i.e. C5, was chosen for mass spectrometric analysis. LC-MS analysis was carried out in order to identify the phytochemicals in CT seed extract with a possible role in interaction with the standard phenolic phytochemicals. In order to retain maximum structural information, a constant value of collision energy was given to each compound for obtaining mass spectra with different fragmentation patterns. The different m/z values were analysed using the RIKEN ReSPect database which is extensively designed to study the mass spectra of the plant based secondary metabolites.

**Figure 3:**
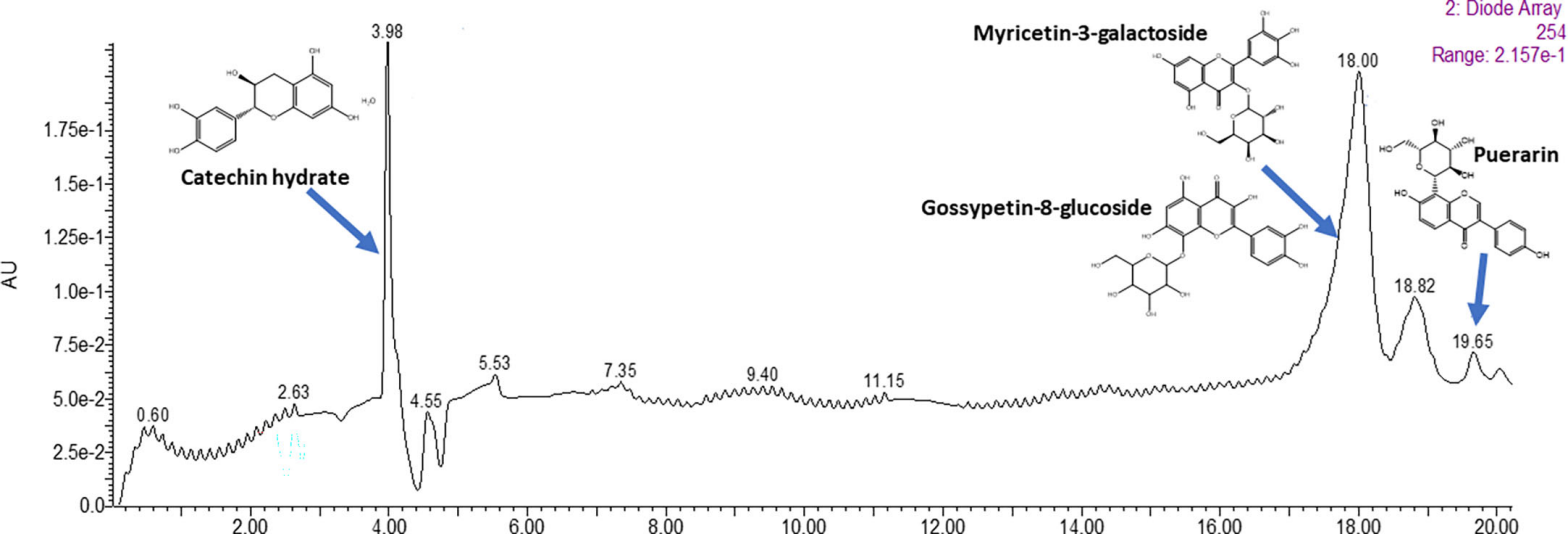
LC-MS chromatogram of purified fraction (C5) of *C. tetragonoloba* seeds obtained after Sephadex LH-20 chromatography.

The gradient flow method was chosen to separate the flavonoids in liquid chromatography. The significant peaks eluted out had the retention times of 3.98 min, 18.0 min, and 19.65 min respectively (**Figure 3**). These peaks were then subjected to ESI-MS full scan mode analyses in order to identify the protonated ions. The individual m/z spectra are given in **Supplementary Material** (**Figure S3**).

Major peak 1 eluted out at 3.98 minutes showed fragments with m/z of values 305.1310 indicating the presence of dihydroflavonol catechin hydrate. The second major peak, which was eluted out at 18.00 minutes showed the fragment with m/z observed at 481.472 indicated the presence of flavonols, Myricetin-3-Galactoside and Gossypetin-8-glucoside. The third major peak at 19.65 minutes indicated the presence of Puerarin which is daidzein 8-C-glucoside belonging to isoflavonoid class and was identified *via* the m/z fragments of 268.0568 and 297.0857. The retention time, chemical formula and mass of individual compounds are given in the **Supplementary Material** (**Table S1**).

In the present work, it has been seen that in comparison to the extract alone, both the *C. tetragonoloba* seed extract and kaempferol showed high fold increase in the antioxidant activity when combined at lower concentrations (**Figure 1**). This could be due to the presence of catechin and myricetin in the seed extract. We assume that the aglycone version of the identified compounds could be involved in synergistic interactions due to available and reactive hydroxyl groups. Many flavonoids are present as aglycones and convert to glycosides as the fruit matures on the plant. Again, upon consumption, the glycosides in food are cleaved into aglycones which are often more bioactive than the glycosylated versions. However, antioxidant synergy with flavonoid glycosides, such as quercetin-3-glucoside, has also been reported (Hidalgo et al., 2010). The authors mentioned that kaempferol showed synergistic interaction with catechin and myricetin.

Here, the chromatogram obtained was according to the polarity of the compounds eventually detected by MS analysis. The analysis showed that the methanolic extract contains only one isoflavonoid viz. puerarin, which was present in its glycosylated form. At relatively less abundance, as observed from the MS count ion, of Vitexin, which is a flavone, was also detected in the extract. The neutral ion losses from the different compounds indicated that the phytochemical constituents in the pods were present in glycosylated as were their aglycone forms. Kobeasy and El-salam (2011) investigated the major flavonoids present in the *C. tetragonoloba* collected from the Egypt region and showed the presence of luteolin and quercetin in aqueous extracts of seeds. Additionally, Morris and Wang (2017) explored the potential of *C. tetragonoloba L*. beans as a good source of kaempferol and quercetin in different cultivars grown in Georgia, USA under laboratory conditions.

To summarize, in the current study, a novel attempt at using *C. tetragonoloba* seed extract to enhance antioxidant activity of polyphenolic phytochemicals (of dietary importance) was made using both cell-free and cell culture systems, followed by purification of phenolics/flavonoids from the seeds (along with detailed phytochemical characterization using LC-MS technique).

A representative graphical abstract of the overall work done is given in **Supplementary Material** (**Figure S1**).

## Conclusion

The current study sought to investigate the antioxidant activity enhancer effect of an edible desert legume, *Cyamopsis tetragonoloba*. Although the seeds of this plant have been used as a commercial source of guar gum, they remain relatively underutilized as sources of nutraceuticals. Furthermore, negligible work has been done towards sourcing antioxidant activity enhancers (AAE) from such edible legumes. The post-COVID era has witnessed a boost in the global nutraceutical industry. Consumers have developed preference towards nutraceuticals and dietary supplements obtained from plants due to lower toxicity. On the basis of the results of this study, it could be concluded that phenolic compounds present in *C. tetragonoloba* seed extract can interact with other compounds (standard phytochemicals) and act as antioxidant activity enhancers. These interactions can be synergistic, additive, or antagonistic, based on various characteristics, like chemical structures, availability of hydrogen ions, type of antioxidant assay used, concentrations, and combination ratios. The results obtained support our hypothesis of edible legumes as a host for a variety of natural antioxidant activity enhancers. Furthermore, the use of legumes as food ingredients and nutraceuticals is extremely promising for developing functional foods with positive health effects, often attributed to the antioxidant potential. The use of edible legume plants growing in the wild in Indian (semi) arid regions in formulating these nutraceuticals can be beneficial from both economic and environmental aspects as these plants are capable of growing on marginal and less fertile lands, and do not need heavy application of water or fertilizers. Being edible and safe, the seed extract of *C. tetragonoloba* can be used in food industry as an antioxidant activity enhancer. Those plant cultivars which are less useful as sources of guar gum (yield- or quality-wise), or even degummed seeds or guar gum industrial waste, could be attractive candidates for the same. The current study would potentially pave the way for more such research towards desert plants as sources of antioxidants/antioxidant activity enhancers, as well as mechanistic elucidation of biological activities of specific ‘plant extract- phytochemical’ combinations.

## Data availability statement

The original contributions presented in the study are included in the article/**Supplementary Material**. Further inquiries can be directed to the corresponding author.

## Author contributions

PD and PS contributed to conception and design of the study. TJ, SM, SP and VA performed the experiments. TJ wrote the first draft of the manuscript. SM, SP and VA wrote sections of the manuscript. All authors contributed to the article and approved the submitted version.
