# Supplementary material for: Investigating the antioxidant activity enhancer effect of Cyamopsis tetragonoloba seed extract on phenolic phytochemicals
Source: Front Plant Sci. 2023 Mar 8;14:1131173. doi: 10.3389/fpls.2023.1131173 (PMC10030946; doi:10.3389/fpls.2023.1131173)
Supplement: Supplementary file 1 [file DataSheet_1.pdf]

Supplementary Material

Investigating the antioxidant activity enhancer effect of *Cyamopsis tetragonoloba* seed extract on phenolic phytochemicals

Tripti Joshi, Sumit Kumar Mandal, Sonakshi Puri, Vidushi Asati, P.R. Deepa and Pankaj Kumar Sharma*

Address: Department of Biological Sciences, Birla Institute of Technology and Science (BITS), Pilani Campus, Pilani, (Rajasthan) 333031, India

*** Corresponding author: pankajsharma@pilani.bits-pilani.ac.in**

Figure S1: Graphical abstract of the reported work

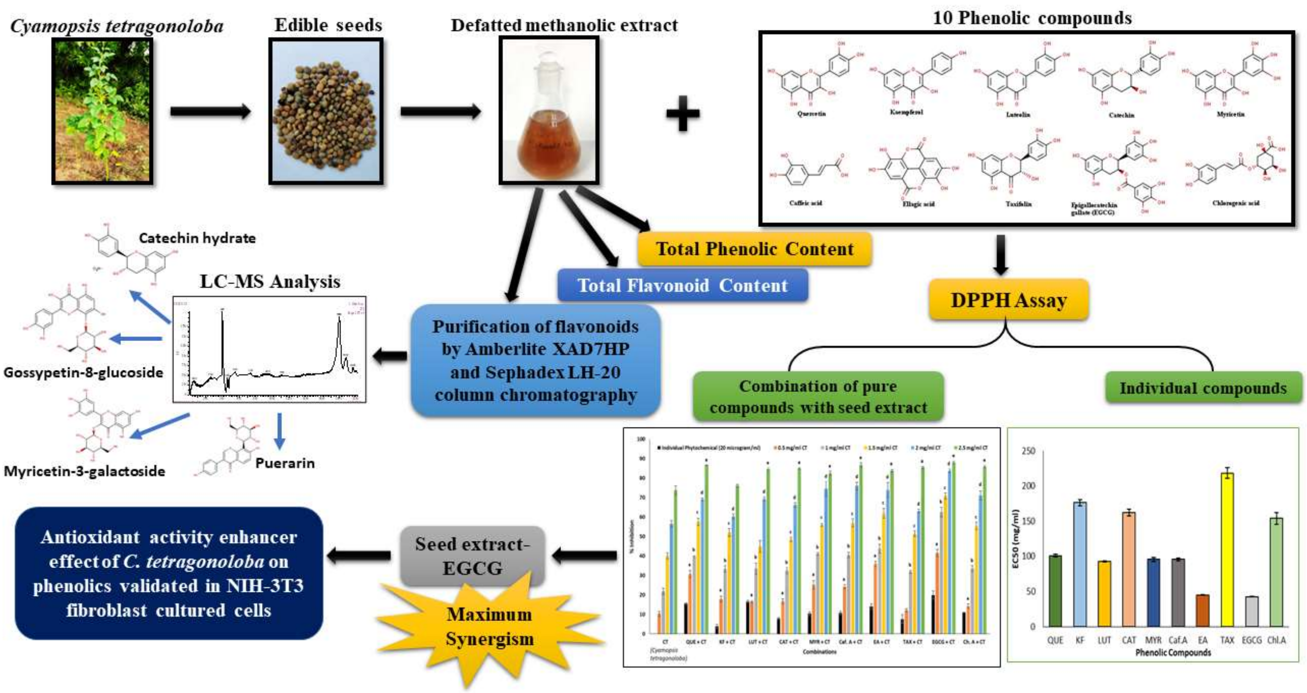

Figure S2: Silica gel F254 TLC analysis of the *C. tetragonoloba* fractions eluted from Sephadex LH20 (Lanes 1-8). D, G, Q represent Daidzein, Genistein and Quercetin, respectively. The solvent system used was Toluene:Acetic Acid:Acetone:Formic Acid :: 20:4:2:1. Image represents illumination at 254 nm.

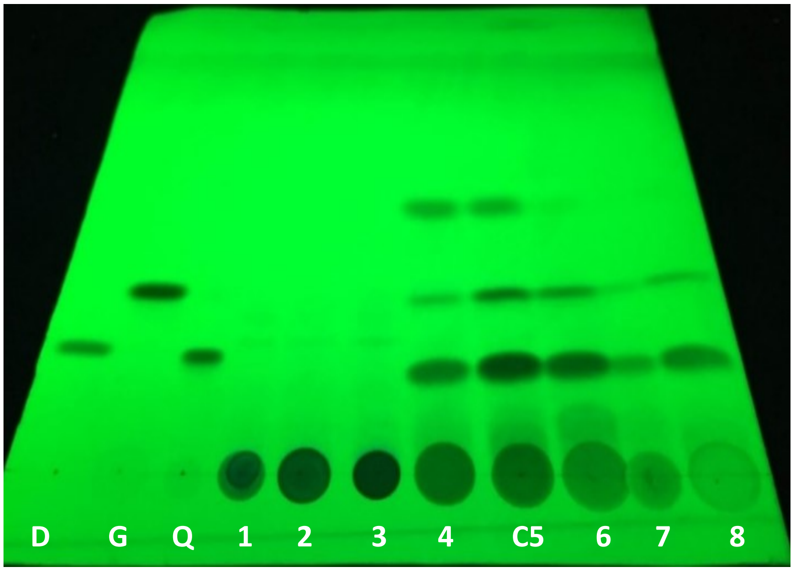

Figure S3: The following are the mass spectra of the peaks depicted in Figure 3. LC-MS was performed at SAIF, Panjab University, Chandigargh, India. The conditions have been mentioned in the main text (Protocol as per Chaudhary et al., 2020; Venuprasad et al., 2014).

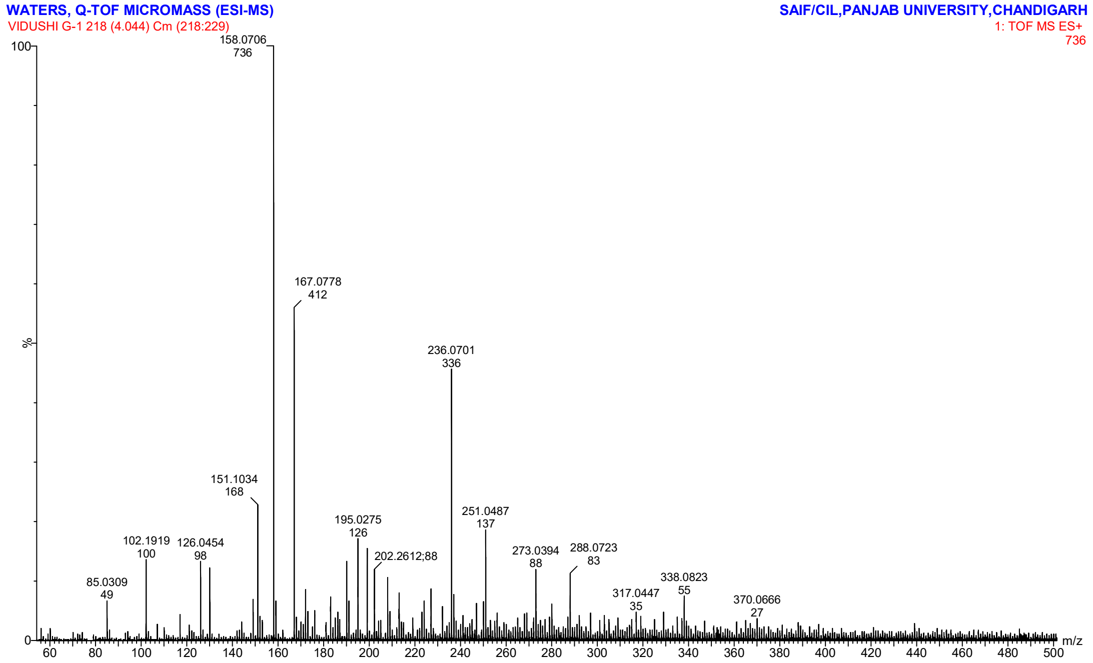

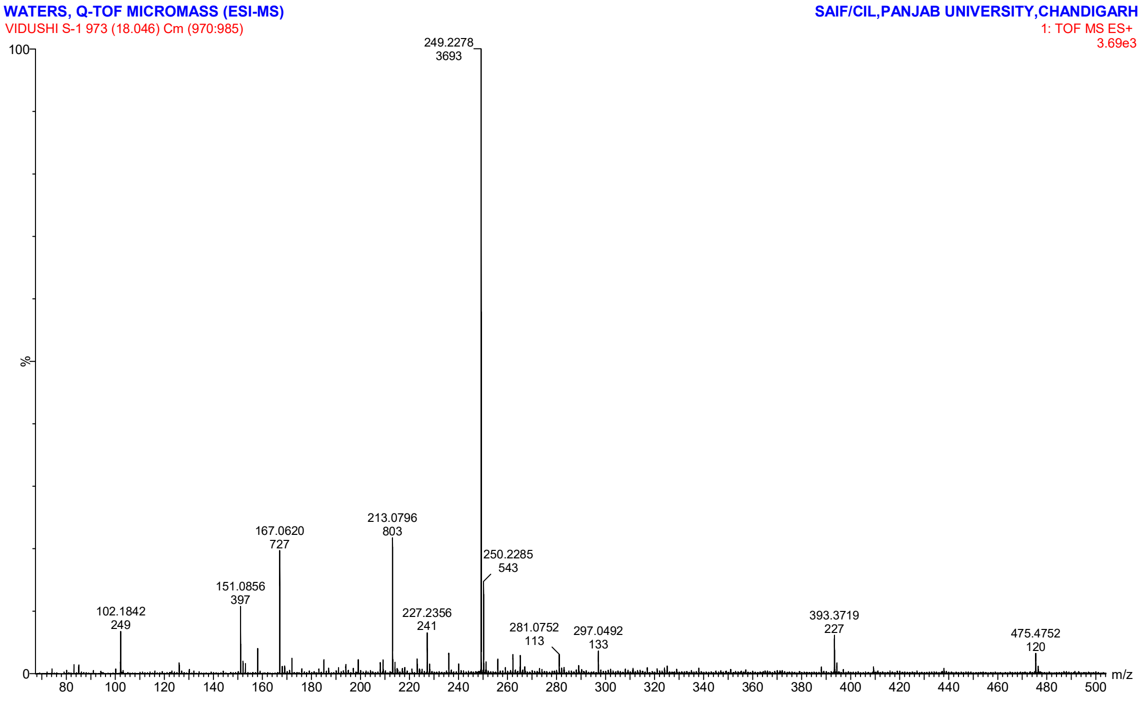
WATERS, Q-TOF MICROMASS (ESI-MS)
VIDUSHI S-1 973 (18.046) Cm (970:985)
SAIF/CIL,PANJAB UNIVERSITY,CHANDIGARH
1: TOF MS ES+
3.69e3
249.2278 3693
213.0796 803
167.0620 727
250.2285 543
151.0856 397
102.1842 249
227.2356 241
393.3719 227
297.0492 133
475.4752 120
281.0752 113
%
m/z

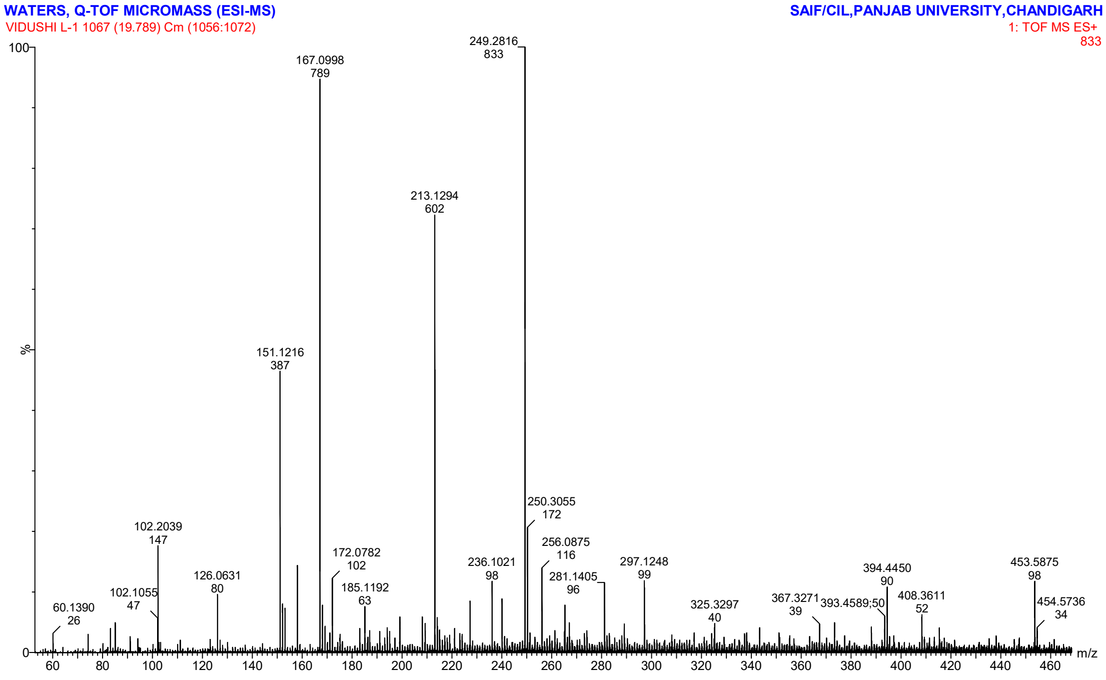
WATERS, Q-TOF MICROMASS (ESI-MS)
VIDUSHI L-1 1067 (19.789) Cm (1056:1072)
SAIF/CIL,PANJAB UNIVERSITY,CHANDIGARH
1: TOF MS ES+
833
249.2816 833
167.0998 789
213.1294 602
151.1216 387
250.3055 172
102.2039 147
256.0875 116
172.0782 102
236.1021 98
281.1405 96
297.1248 99
453.5875 98
394.4450 90
126.0631 80
185.1192 63
408.3611 52
102.1055 47
325.3297 40
367.3271 39
393.4589;50
454.5736 34
60.1390 26
%
m/z

Table S1. MS analysis of *Cyamopsis tetragonoloba* seed phenolics (done for C5 fraction eluted from Sephadex LH20 column). The $[M+H]^+$ molecules of the flavonoids/isoflavonoids detected in the purified extract, their retention time and structures are represented. All the structures were derived from ChemSpider database. The analysis was done using RIKEN-ReSpect software [Sawada Y, et al. (2012). RIKEN tandem mass spectral database (ReSpect) for phytochemicals: a plant-specific MS/MS-based data resource and database. Phytochemistry, 82, 38-45. doi: 10.1016/j.phytochem.2012.07.007].

S. No.	Retention time (min)	Molecular weight	[Typical MS/MS Ions] (m/z)	Formula	Compound identification	Structure	Category
1.	3.98	308.28	305.1310<br>288.0723<br>158.0706	$C_{15}H_{12}O_7$	Catechin hydrate	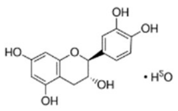	Dihydroflavonol
2.	18.00	480.4	481.472<br>151.0856	$C_{21}H_{20}O_{13}$	Myricetin-3-Galactoside	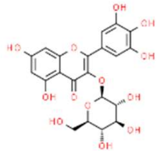	Flavonol
3.		480.4	481.472<br>167.0620<br>213.0796	$C_{21}H_{20}O_{13}$	Gossypetin-8-glucoside	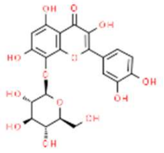	Flavonol
4.	19.65	417.5	268.0568<br>297.0857	$C_{21}H_{20}O_9$	Puerarin (daidzein-8-C-glucoside)	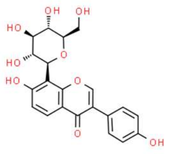	Isoflavonoid
